# Role of mTOR Complex 1 Signaling Pathway in the Pathogenesis of Diabetes Complications; A Mini Review

**DOI:** 10.22088/IJMCM.BUMS.10.3.181

**Published:** 2022-01-10

**Authors:** Amir Yarahmadi, Negar Azarpira, Zohreh Mostafavi-Pour

**Affiliations:** 1 *Department of Biochemistry, School of Medicine, Shiraz University of Medical Sciences, Shiraz, Iran.*; 2 *Department of Clinical Biochemistry, Faculty of Medicine, Mashhad University of Medical Sciences, Mashhad, Iran.*; 3 *Transplant Research Center, Shiraz University of Medical Sciences, Shiraz, Iran.*; 4 *Autophagy Research Center, Shiraz University of Medical Sciences, Shiraz, Iran.*

**Keywords:** Diabetes, complications, mTOR, mTOR complex 1, signaling

## Abstract

The mammalian target of rapamycin (mTOR) is an evolutionarily conserved serine/threonine-protein kinase that senses and combines various environmental signals to regulate the growth and homeostasis of human cells. This signaling pathway synchronizes many critical cellular processes and is involved in an increasing number of pathological conditions such as diabetes, cancer, obesity, and metabolic syndrome. Here, we review different complications of diabetes that are associated with mTOR complex 1 imbalance. We further discuss pharmacological approaches to treat diabetes complications linked to mTOR deregulation.

Diabetes mellitus is a multifactorial disease characterized by high blood glucose concentration, and has become a significant health problem in developing countries (1, 2). It has been associated with many human diseases such as cancers, cardiovascular, renal, and blood vessel failure (1,3,4). Marked high blood glucose (hyperglycemia) causes main symptoms of diabetes including polyuria, polydipsia, and polyphagia (1). American diabetes association classifies diabetes mellitus in two different forms known as type 1 or insulin-dependent diabetes mellitus and type 2 or non-insulin-dependent diabetes mellitus (1). Previous studies showed that the mammalian target of rapamycin (mTOR) signaling pathway has an essential role in the pathogenesis of metabolic syndrome, obesity, and diabetes (5). The mTOR is an evolutionarily well-conserved serine/threonine-protein kinase that serves as a critical regulator of cell metabolism, proliferation, growth, and survival (6-9). Increased mTOR activity is common in most human diseases such as cancers, diabetes, and genetic disorders (5,10, 11).

The mTOR kinase can form two distinct multi-protein complexes named mTOR complex 1 (mTORC1) and mTOR complex 2 (mTORC2) (12).

mTORC1 consists of 6 components, including the catalytic mTOR subunit, regulatory-associated protein of mTOR (RAPTOR), the mammalian lethal with sec-13 protein-8 (mLST8 or GBL), the DEP domain-containing mTOR-interacting protein (DEPTOR), the Tti1/Tel2 complex, and the proline-rich Akt substrate 40 kDa (PRAS40) (5, 10). mTORC1 promotes the phosphorylation of two downstream proteins, ribosomal S6 kinase 1(S6K1) and eukaryotic translation initiation factor 4E (eIF4E)-binding protein (4E-BP1) which leads to crucial cellular processes such as transcription, translation, protein, and lipid synthesis, cell growth and metabolism (Figure 1) (7). mTORC1 responses to amino acids, stress, glucose, growth factors such as insulin and insulin-like growth factor 1 (IGF-1) (13).

mTORC2 has seven subunits, four similar with mTORC1: mTOR, DEPTOR, Tti1/Tel2, and mLST8 and three specifics for mTORC2: rapamycin-insensitive companion of mTOR (RICTOR), mammalian stress-activated MAP kinase-interacting protein1 (mSIN1), and protein observed with rictor 1 and 2 (PROTOR1/2) (14). The main downstream targets of mTORC2 are all AGC subfamily kinases, including Akt (PKB), serum- and glucocorticoid-induced protein kinase 1 (SGK1), and a protein kinase that regulates cell survival, migration, metabolism, and cytoskeletal organization (15).

mTORC1 controls glucose homeostasis in many tissues such as the liver, fat (adipose), β-cells, and skeletal muscle through serine phosphorylation of insulin receptor substrate 1 (IRS-1) via mTORC1/S6K1 activity (16). Also, mTORC1plays a vital role in the regulation of the β-cell size/mass and function, which are essentialin the pathogenesis of diabetes mellitus (7,16, 17).

**Fig.1:**
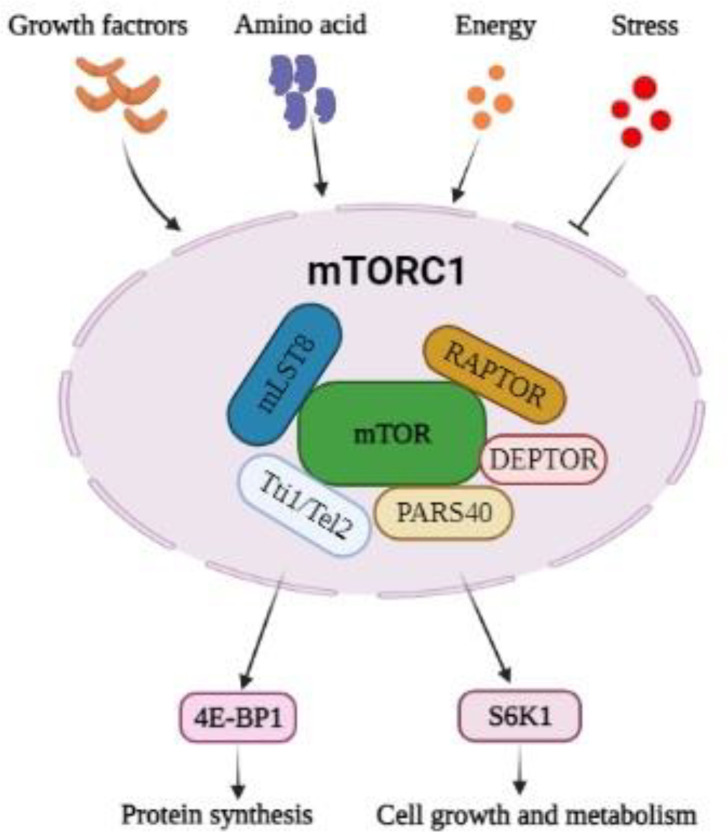
**General composition of the mTOR complex 1 and its importance in cellular growth and metabolism.** mTOR: mammalian target of rapamycin; RAPTOR: regulatory-associated protein of mTOR; mLST8: mammalian lethal with sec-13 protein-8; DEPTOR: DEP domain-containing mTOR-interacting protein; PRAS40: proline-rich Akt substrate 40 kDa; 4E-BP1: eukaryotic translation initiation factor 4E-binding protein; S6K1: protein S6 kinase 1.

Because of mTORC1 ability to integrate insulin and nutrients responses and its impact on glucose homeostasis, it seems necessary to understand more about its biological effects during diabetes mellitus. This review summarizes major findings and the latest information regarding the role of the mTORC1 signaling pathway in the pathogenesis of diabetes complications and suggests the potential pharmacological approaches to treat diabetes complications linked to mTOR deregulation.

**mTORC1 in insulin secretion and glucose home-ostasis**

β-cells located in the pancreas secrete insulin in response to many nutrients, and have an essential impact on the regulation of glucose homeostasis (5, 18). Temporary activation of mTORC1 results in expansion of β-cell size, mass and insulin production, while constitutive activation of mTORC1 showed contradictory results (19, 20). mTORC1 signaling is a positive regulator of β-cells mass and functions in response to nutrients (21). For instance, in mice, constitutive activation of mTORC1 in the β-cells of the pancreas declines blood glucose, increases insulin secretion, and positively impacts glucose tolerance (5). Conversely, in the experiments in which glucose or IGF-1 is used to stimulate mTORC1 in β-cells, the IRS2/Akt pathway inhibition leads to β-cell apoptosis and glucose intolerance (22). Also, inhibition of mTORC1 by rapamycin exacerbates hyperglycemia in type 2 diabetes, showing the importance of mTORC1 in the function of pancreas (23, 24). Similarly, in the liver mTORC1 signaling pathway influences systemic glucose and insulin homeostasis (25). Permanent activation of the mTORC1 signaling pathway shows intense inhibitory effects on IRS-1, which decline the Akt signaling pathway. This phenomenon causes an imbalance between the liver glycolysis pathway and glucose uptake from the blood, and results in glucose intolerance (25, 26).

**Role of mTORC1 in obesity**

Obesity is a hazardous risk factor for the development of diabetes. Obesity may be observed with chronic systemic inflammation through excess fat tissue accumulation with necessary calorie exceeding energy saving (27). Hyperinsulinemia and insulin resistance are more common among obese patients and are related to a poor prognosis in diabetes (28). A growing body of evidence shows that the mTOR pathway is strongly involved in initiating and developing obesity and insulin resistance in metabolic syndrome (29, 30). mTOR signaling pathway is crucial for adipogenesis, and rapamycin interfers with the proliferation and differentiation of human adipocyte differentiation in primary culture cells (31). Furthermore, Chang et al. showed that rapamycin decreased obesity induced by a high-fat diet in mice via long-term inhibition of mTORC1 (32).

**mTORC1 and lipid metabolism**

mTORC1 plays a crucial role in promoting lipogenesis by modulating the expression of many lipogenic genes (33). A significant family of transcription factors that regulate lipid synthesis is the sterol regulatory element binding proteins (SREBPs) (33, 34). SREBPs belong to the family of basic helix-loop-helix-leucine zipper (bHLH-Zip) transcription factors. The SREBPs family has three closely related members: SREBP1a, SREBP1c, and SREBP2 (35,36). mTORC1 stimulates the movement, processing, and transcription of SREBPs. SREBPs adjust lipid homeostasis by regulating the expression of various enzymes necessary for endogenous cholesterol, fatty acid (FA), triacylglycerol, and phospholipid synthesis. SREBP-1c is required for FA synthesis and insulin-induced glucose metabolism (especially in lipogenesis). In contrast, SREBP-2 is more specific to cholesterol synthesis. The SREBP-1a isoform seems to be involved in both pathways (36). *In vivo* studies have shown that mice deficient for mTORC1 in their liver, through raptor knockout in their liver, not only are not able to induce Srebp1c and lipogenesis but also have decreased levels of both liver triglycerides and cholesterol on a Western diet (37-40). These fundamental roles of mTORC1 in lipid metabolism make it a suitable target for reducing lipids synthesis during diabetes (41).

**Role of mTORC1 in diabetic nephropathy**

Diabetic nephropathy is a significant cause of end-stage kidney disease, and a major health problem around the world (8). A primary complication of diabetic nephropathy is proteinuria caused by the destruction of the glomerular filtration barrier in podocytes (42). Inhibition of mTORC1 has been reported to cause proteinuria in different patients (43). mTOR function in podocytes is critical for the integrity of the filtration barrier (44). Different studies reported that the administration of sirolimus prevents the development of diabetic nephropathy in mouse models of both type 1 and type 2 diabetes. For instance, Inoki et al. found that mTORC1 activation was involved in many molecular events in podocytes, consisting of ER stress with a fibroblastic phenotypic change that leads to podocyte injury and proteinuria in mice (45). Furthermore, Gödel et al. proved that normal activation of mTORC1 has a positive function in podocyte participation in glomerular expansions for kidney development, and hyperactivation of mTORC1 will be accompanied by podocytes dysfunction and progression of diabetic nephropathy (46).

**Role of mTORC1 in the pathogenesis of diabetic retinopathy**

Diabetic retinopathy remains one of the most common leading causes of vision impairment in the world, and it is a significant consequence of prolonged diabetes (47). Retinal microvascular defects and enhanced protein degradation are the leading cause of retinopathy during uncontrolled hyperglycemia. Activation of the PI3K/Akt/mTOR signaling pathway has been linked to impaired glucose metabolism in retinal tissue (48, 49). A recent study suggests that Akt's interaction with Ras homolog gene family member B (RhoB) promotes endothelial cell survival and development during vascular genesis, which probably can lead to angiogenesis characteristic of diabetic microva-scular disease (50). Therefore, it can be concluded that suppression of the PI3K/Akt/mTOR signaling pathway interrupts Akt-RhoB interaction, increases endothelial cell death, and will help prevent diabetic retinopathy. Stopping endothelial cell proliferation and inducing apoptosis can be considered a treatment model to prevent vascular abnormalities, which has been seen in diabetic retinopathy (51, 52). Also, it has been shown that inflammation and oxidative stress have a significant role in diabetic retinopathy (53, 54). In diabetic conditions, in the retina, advanced glycation end products (AGEs) generate oxidative stress, promote changes in proteins, and enhance the level of inflammatory cytokines that make changes to vascular function (55). A growing body of evidence implies that existing inflammatory processes within the retina make it more susceptible to the progression of diabetic retinopathy (56).

**Relation between mTORC1, diabetes, and in-flammation**

Previous studies revealed the connection between inflammation and diabetes (57). The mTOR signaling pathway can be activated by various ligands such as glucose, growth factors, amino acids, and nutrients. Moreover, inflammatory stimuli, while attached to antigen receptors, cytokine, or toll-like receptors (TLRs), can also activate mTORC1 in the cells. At the molecular level, mTOR potentially regulates the activity of inflammatory transcription factors such as nuclear factor kappa B (NF-κB), signal transducer and activator of transcription–3 (STAT3), and some interferon regulatory factors in a cell-type-specific algorithm (58). In normal conditions, tyrosine phosphorylation of insulin receptor substrate (IRS) proteins can activate the PI3K-Akt-mTOR pathway, resulting in normal insulin response in the cells. Serine threonine phosphorylation of IRS will cause negative regulation of insulin signaling pathways, and during this condition, insulin resistance happens in the body (59). Downstream effector of mTORC1, S6 kinase (S6K) also phosphorylates IRS proteins resulting in insulin resistance in the cells. During inflammation, activation of mTORC1 and its effector S6K leads to phosphorylation of IRS-1 and insulin resistance, an important phenomenon in the pathophysiology of diabetes. The inhibition of mTORC1 with rapamycin can potentially reduce S6K activation, and could be used to treat insulin resistance in diabetes (60). Recent studies have shown that the mTOR signaling pathway plays a crucial role in regulating pro- and anti-inflammatory responses in immune cells (56). During type 1 diabetes, autoimmune destruction of beta-cells of the pancreas takes place. So, it is important to consider the mTOR signaling pathway as a therapeutic target in the treatment of type 1 diabetes (61). 

**mTORC1 and oxidative stress**

Oxidative stress has been defined as an imbalance between increased reactive oxygen species (ROS) generation and reduced antioxidant defense systems in the body (62). ROS are increased during hyperglycemia and can damage different organs. Increased intracellular ROS can also trigger several pro-inflammatory pathways and cytokine production, activating mTORC1 and its effector S6K. Activated S6K phosphorylates IRS-1 proteins and results in insulin resistance in the cells during diabetes (63). Also, it has been reported that high glucose level induces ROS formation in the glomerular mesangial cells, and leads not only to a decline of the antioxidant enzyme activity and glutathione (GSH) level, but also promotes NADPH oxidase activity, and increases the expression of *P53* and Bax/Bcl-2 ratio resulting in apoptosis promotion (64). Treatment of the mesangial cells via rapamycin reduced oxidative stress and apoptosis in the cells exposed to high glucose, and ameliorated the effects of mTOR activation, which improved the complication of diabetic nephropathy (64, 65). This data shows that mTOR plays a key role in modulating ROS-induced oxidative stress in mesangial cells during diabetes (Figure 2).

**How to quench mTOR to prevent diabetes complications**

Rapalogs (including rapamycin and everol-imus) are the first generation of mTOR inhibitors and were first used as immunosuppressive drugs to avoid transplantation rejection and then be used in targeted anti-cancer kinase inhibitors are the second generation of mTOR inhibitors based on their activity to inhibit mTOR (69). Because of sequence similarity between mTOR and PI3K, ATP competitive PI3K inhibitors are now found to have different degrees of mTOR inhibitory effects. However, it has been proved that a selective mTOR inhibitor is better than dual function inhibitors because of many different functions of diverse isoforms of PI3K (16, 66)**.** Two main mTOR inhibitors are sirolimus (rapamycin) and everolimus that have substantial inhibitory effects on the mTOR signaling pathway that may be beneficial in reducing diabetes complications. However, their use is restricted because of some adverse effects; therefore, it is necessary to do more studies about the clinical application of these drugs (67).

**Fig.2:**
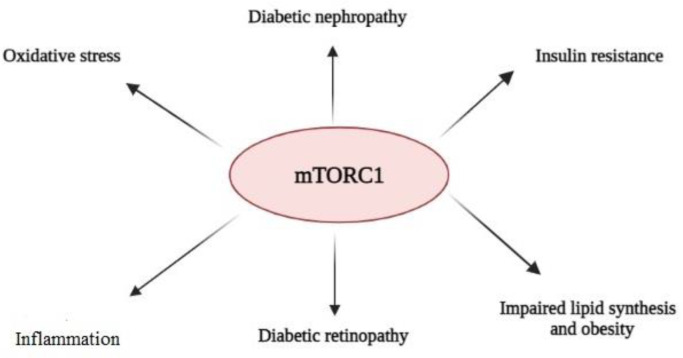
Role of mTOR complex 1 in diabetes complications

## Conclusions and future perspectives

This review explained the significance of the mTORC1 signaling pathway in the pathophy-siology of diabetes complications regarding the importance of mTORC1 in many cellular processes involved in diabetes complications. It seems necessary to develop more studies about the mTORC1 signaling pathway to better understanding the molecular mechanisms of development of metabolic diseases such as diabetes.

## Conflicts of interest

The authors declare that there is no conflict of interest.
